# Activated Protein Synthesis and Suppressed Protein Breakdown Signaling in Skeletal Muscle of Critically Ill Patients

**DOI:** 10.1371/journal.pone.0018090

**Published:** 2011-03-31

**Authors:** Jakob G. Jespersen, Anders Nedergaard, Søren Reitelseder, Ulla R. Mikkelsen, Kasper J. Dideriksen, Jakob Agergaard, Frederik Kreiner, Frank C. Pott, Peter Schjerling, Michael Kjaer

**Affiliations:** 1 Department of Orthopedic Surgery M, Institute of Sports Medicine Copenhagen, Bispebjerg Hospital and Center for Healthy Aging, Faculty of Health Sciences, University of Copenhagen, Copenhagen, Denmark; 2 Department of Rheumatology, Institute of Inflammation Research, Rigshospitalet, Copenhagen, Denmark; 3 Department of Anesthesiology, Bispebjerg Hospital, Copenhagen, Denmark; Ohio State University, United States of America

## Abstract

**Background:**

Skeletal muscle mass is controlled by myostatin and Akt-dependent signaling on mammalian target of rapamycin (mTOR), glycogen synthase kinase 3β (GSK3β) and forkhead box O (FoxO) pathways, but it is unknown how these pathways are regulated in critically ill human muscle. To describe factors involved in muscle mass regulation, we investigated the phosphorylation and expression of key factors in these protein synthesis and breakdown signaling pathways in thigh skeletal muscle of critically ill intensive care unit (ICU) patients compared with healthy controls.

**Methodology/Principal Findings:**

ICU patients were systemically inflamed, moderately hyperglycemic, received insulin therapy, and showed a tendency to lower plasma branched chain amino acids compared with controls. Using Western blotting we measured Akt, GSK3β, mTOR, ribosomal protein S6 kinase (S6k), eukaryotic translation initiation factor 4E binding protein 1 (4E-BP1), and muscle ring finger protein 1 (MuRF1); and by RT-PCR we determined mRNA expression of, among others, insulin-like growth factor 1 (IGF-1), FoxO 1, 3 and 4, atrogin1, MuRF1, interleukin-6 (IL-6), tumor necrosis factor α (TNF-α) and myostatin. Unexpectedly, in critically ill ICU patients Akt-mTOR-S6k signaling was substantially higher compared with controls. FoxO1 mRNA was higher in patients, whereas FoxO3, atrogin1 and myostatin mRNAs and MuRF1 protein were lower compared with controls. A moderate correlation (r^2^ = 0.36, p<0.05) between insulin infusion dose and phosphorylated Akt was demonstrated.

**Conclusions/Significance:**

We present for the first time muscle protein turnover signaling in critically ill ICU patients, and we show signaling pathway activity towards a stimulation of muscle protein synthesis and a somewhat inhibited proteolysis.

## Introduction

Skeletal muscle, which constitutes 30–40% of human body weight [1], is critical for numerous functions such as breathing, locomotion and metabolism, and this most abundant tissue also serves as a large protein reservoir. Loss of muscle mass is a hallmark of various diagnoses like cancer, chronic heart failure, chronic obstructive pulmonary disease and HIV-infection, and weight loss or low fat free mass independently predicts mortality in these conditions [2], [3]. Likewise, debilitating muscle atrophy is part of critical illness [4], [5], [6], [7], [8] occurring at a mean rate of 3–4% of fiber cross-sectional area per day [5]. Skeletal muscle mass is regulated through the balance between the rates of protein synthesis and protein breakdown. The signaling pathways mediating this balance include, but are not limited to, the Akt-mTOR, Akt-GSK3β, Akt-FoxO and the myostatin pathways [9], [10], [11]. Akt (aka protein kinase B) is activated by insulin and IGF-1 and has two known major downstream branches relevant to muscle protein synthesis: the mTOR pathway, which is activated by Akt, and the GSK3β pathway, which is inhibited by Akt. mTOR seems to be a key regulator of cell size and protein synthesis combining signals from growth factors and nutrients [9], [11]. mTOR is comprised of complex1 and 2 (mTORC1 and mTORC2), and the effect of mTOR on protein synthesis is mediated through mTORC1 by the activation of S6k and the inhibition of 4E-BP1. Akt also phosphorylates and inactivates the FoxO transcription factor family, which consists of FoxO1, 3, 4 and 6 [9]. The activation of FoxO3 causes muscle loss through increased proteolysis, and FoxO3 induces the muscle specific ubiquitin ligases atrogin1/MAFbx (from here on referred to as atrogin1) and MuRF1 [12], [13], [14], [15]. In fact, muscle that lack either of these ligases atrophy less upon disuse [16], and atrogin1, MuRF1 and FoxO1 belong to a set of muscle atrophy-related genes termed “atrogenes” [17]. The transforming growth factor-β (TGF-β) superfamily member myostatin is another negative regulator of muscle mass, and the inhibition of myostatin increases muscle mass, whereas the overexpression of it induces muscle atrophy [18], [19], [20].

Accordingly, myostatin and Akt-dependent signaling on mTOR, GSK3β and FoxO pathways play key roles in the control of skeletal muscle protein turnover, and animal studies have examined the role of these pathways in critical illness models [10], [21], [22], but it is unknown how these pathways are regulated in critically ill human muscle. Thus, the aim of our study was to investigate the Akt-mTOR, Akt-GSK3β and Akt-FoxO signaling pathways as well as expression of TGF-β members in skeletal muscle of critically ill ICU patients. We hypothesized that ICU patients would have higher proteolytic and lower protein synthesis signaling compared with controls.

## Materials and Methods

### Subjects and ethics

The experimental group consisted of 12 patients admitted to the Intensive Care Unit at Bispebjerg Hospital, Copenhagen University Hospital. The control group consisted of 12 age- and sex-matched healthy, ambulatory controls. Since the transcriptional profile of human critical illness skeletal muscle agrees poorly with that of inactivated skeletal muscle [23] the use of ambulatory controls is justified. Exclusion criteria were being younger than 18 years, pregnant or nursing. Informed and surrogate informed written consent was obtained from control subjects and relatives to patients, respectively. The study followed the rules of the Declaration of Helsinki and was approved by the local ethical committee of Copenhagen and Frederiksberg municipality (KF 01 260751).

As part of their clinical treatment the ICU patients received intravenous insulin therapy to control critical illness hyperglycemia, and at the time when they were recruited the local ICU insulin therapy reflected “intensive insulin therapy” [24], [25]. Insulin (Novorapid, Novo Nordisk, Bagsvaerd, Denmark) infusion was adjusted with the aim of maintaining normoglycemia based on measurements of whole blood glucose in undiluted arterial blood, performed at one-to four-hour intervals with the use of a glucose analyzer (ABL700, Radiometer Medical, Copenhagen, Denmark). All patients received enteral (Fresubin Original, Fresenius Kabi, Bad Homburg, Germany) and/or parenteral (StructoKabiven, Fresenius Kabi) nutrition.

### Blood analyses

Blood levels of leukocytes, C-reactive protein (CRP), urea and creatinine were determined in-house (ISO certified lab), and the stated numbers derive from measurements at 06:00 A.M. at the day of the biopsy. Multiplex assays performed according to the manufacturer's instructions (HSCYTO60SK or MPXHCYTO-60K, Milliplex, Millipore, Billerica, MA, USA) were used to measure levels of the systemic inflammatory markers TNF-α, IL-6, IL-8, IL-10 and monocyte chemoattractant protein-1 (MCP-1)/chemokine (C-C motif) ligand 2 (CCL2) in blood sampled at the time of the biopsy. Blood samples from controls were taken at rest in the morning. Plasma amino acid concentrations were quantified by HPLC as described recently by our group [26]. Plasma IGF-1 concentrations were determined by a time-resolved immunofluorometric assay (Perkin Elmer, Turku, Finland) [27].

### Muscle biopsies

Muscle biopsies were obtained under local Lidocaine anesthesia from the vastus lateralis muscle (m. quadriceps femoris) using the percutaneous needle biopsy technique of Bergström [28] with 5 mm biopsy needles and manual suction. On extraction, the biopsy was divided and immediately frozen in liquid nitrogen. Biopsies were stored at -80°C pending analyses.

### RNA extraction

Approximately 10 mg of frozen muscle tissue was homogenized in a microvial containing 1 silicium carbide crystal, 5 steel beads (2.3 mm) and 1000 µl TRI-reagent (Molecular Research Center, Cincinnati, OH, USA) using a FastPrep-24 (MP Biomedicals, Solon, OH, USA) homogenizer. Each sample was shaken 3 times in the homogenizer for 15 s at speed 4 with a 2 min interval between each run, in which samples were kept in ice-water bath. Total RNA was extracted according to Chomczynski & Sacchi [29]. After homogenization, 100 µl 1-bromo-3-chloropropane was added to separate the samples into an aqueous and an organic phase. After isolation of the aqueous phase, RNA was precipitated using isopropanol, washed in ethanol and subsequently dissolved in RNase-free water. RNA concentrations were measured by spectroscopy at 260 nm. Good RNA quality was ensured by spectrophotometer ratios at 260/240 nm (acceptable range 1.2–1.6 at pH 8) and 260/280 nm and by denaturing agarose gel electrophoresis.

### Reverse transcriptase quantitative PCR (RT-qPCR)

Total RNA (750 ng from each muscle sample) was converted into cDNA in 20 µl using the OmniScript reverse transcriptase (Qiagen, Valencia, CA, USA) according to the manufacturer's instructions. For each mRNA target, 0.25 or 0.50 µl cDNA was amplified in a 25 µl SYBR Green PCR reaction containing 1X Quantitect SYBR Green Master Mix (Qiagen) and 100 nM of each primer (Table S1). The amplification was monitored real-time using the MX3005P real-time PCR machine (Stratagene, La Jolla, CA, USA). The threshold cycle (Ct) values were related to standard curves made with cloned PCR products to determine the relative difference between the unknown samples, and the specificity of the PCR products was confirmed by melting curve analysis after amplification. Due to limited amount of tissue n equals 11 in each group for RT-qPCR. TNF-α mRNA was expressed at low levels and in some samples TNF-α mRNA was below the detection limit resulting in 0 values. Thus, TNF-α mRNA was analyzed in quadruplicate to reduce stochastic variation, and the median raw values were used for non-parametric statistical analysis. All mRNA data were normalized to acidic ribosomal protein P0 (RPLP0).

### Western blot

Approximately 30 mg of frozen muscle tissue was homogenized as described for RNA extraction, except that it was done in 500 µl ice-cold homogenization buffer (50 mM Tris-base, 1 mM EDTA, 1 mM EGTA, 10 mM beta-glycerophosphate, 50 mM sodium fluoride, 0.5 mM sodium orthovanadate, 0.1% v/v, Triton-X, 0.1% v/v mercaptoethanol and protease inhibitor (Complete, Roche, Basel, Schwitzerland), pH 7.5). Protein concentrations were determined with the Bradford assay and equal amounts (10 µg/10 µl) was added 2 µl 6X Laemmli buffer (62.5 mM Tris-base (pH 6.8), 2% w/v sodium dodecyl sulphate (SDS), 40% v/v glycerol, 5% v/v mercaptoethanol and 1 mg/ml bromophenol blue), heated at 90°C for 4 min, shortly vortexed, spun for 5 s in a microcentrifuge and separated by SDS-PAGE using a 4-12% Bis-Tris gel (Criterion, Bio-Rad, Hercules, CA, USA) at 200 V for 1 h. Gels were blotted (Trans-blot cell, Bio-Rad, 400 mA, 2 h) to polyvinylidene difluoride membranes (Amersham Hybond LFP, GE Healthcare, Buckinghamshire, UK) in transfer buffer (50 mM Tris-base, 383 mM glycine, 20% v/v methanol), except mTOR total and phospho proteins that were transferred without methanol. Then, membranes were washed (1×5 min) in Tris-buffered saline with 0.1% v/v Tween 20 (TBST, pH 7.4), blocked for 30 min with 20% Odyssey blocking buffer (Li-Cor Biosciences, Lincoln, NE, USA) in phosphate-buffered saline, washed (3×5 min), incubated overnight at 4°C in primary antibody, washed (3×5 min), incubated for 1 h in fluorophore-conjugated secondary antibody, washed (3×5 min), and visualized with the Odyssey Infrared Imaging System (Li-Cor Biosciences). All total and phosphorylated protein pairs, except for mTOR, were detected simultaneously on the same membrane. Equal loading was verified by Coomassie staining. Band intensities were quantified using ImageJ (National Institutes of Health, Bethesda, MD, USA). For details on antibodies see table S2.

### Data presentation and statistical analyses

Data in figures are presented as individual values and means in scatter plots, whereas data in tables are means ± standard error of the mean (SEM). Muscle mRNA and protein data were log-transformed and are presented as individual values relative to control mean. Statistical differences between groups were determined using unpaired, two-tailed, unequal variance Welch's t-tests or for TNF-α mRNA Mann-Whitney U test. The relationship between insulin infusion dose and phosphorylated Akt was evaluated by linear regression analysis. This is biologically relevant as Akt is part of the canonical insulin-phosphatidylinositol 3-kinase (PI3K) signaling pathway [30]. Results were considered significant when p<0.05.

## Results

### Patient characteristics

ICU patient characteristics are provided in table 1. All patients required mechanical ventilation at the time of the biopsy. Patients were 64±5 years old, equally represented both sexes and had diagnoses of pulmonary and/or abdominal origin. 8 patients were septic and 10 patients had pneumonia and/or respiratory insufficiency. 5 patients died in the ICU, whereas 7 survived, of whom one died subsequently at another hospital department (Table 1).

**Table 1:** ICU patient characteristics.

Patient #	Diagnoses	Age/sex	ICU days at biopsy	Survival	Days of ICU stay	Vaso-pressor agents	Sedation	Synthetic glucocorticoids	Substance use	Nutrition
1	Thorax trauma, sepsis	28/M	3	S	22	NA	Propofol, fentanyl	Hydrocortisone 50 mg IV 4/daily	Alcohol, cannabis, tobacco	PN+EN
2	Pneumonia, sepsis, respiratory insufficiency	71/M	16	S	27	None	None	None	Tobacco (Alcohol previously)	EN
3	Intestinal necrosis, sepsis, respiratory insufficiency	56/F	2	D, day 6	6	NA	Propofol, remifentanil	Hydrocortisone 50 mg IV 4/daily	Alcohol, tobacco	PN
4	Perforated peptic ulcer, sepsis, respiratory insufficiency	90/F	3	D, day 21	21	NA	Remifentanil	None	None	PN
5	Pneumonia, respiratory insufficiency	68/M	3	D, day 7	7	NA	Propofol, remifentanil	None	Alcohol, tobacco	EN
6	Intestinal perforation, sepsis, respiratory insufficiency	73/F	17	S	27	NA	Fentanyl	None	Tobacco	EN
7	Pneumonia, respiratory insufficiency	77/F	13 (11)^1^	S	41	NA	None	Prednisolone 37.5 mg PO 1/daily	(Tobacco previously)	EN
8	Pneumonia, respiratory insufficiency, cardiac arrest	68/M	7 (5)^1^	S	14	NA	Propofol, remifentanil	None	Alcohol	PN
9	Liver coma, pneumonia, respiratory insufficiency	61/M	2	D, day 11	11	None	None	None	Alcohol, tobacco	EN
10	Abdominal abscess, sepsis, respiratory insufficiency	47/M	1	D, day 2	2	NA, A	Propofol, remifentanil	Hydrocortisone 100 mg IV 4/daily	(Alcohol previously)	PN
11	Peritonitis, sepsis	71/F	2	S (died day 9 at other dept.)	3	None	Propofol, remifentanil	None	(Tobacco previously)	EN+PN
12	Cardiac arrest, pneumonia, sepsis, respiratory insufficiency	61/F	2	S	4	NA	None	None	Alcohol	EN
Mean ± SEM		64±5	6±2		15±4					
Mean ± SEM controls		65±4								

ICU days at biopsy: Time spent in the ICU at the time of the muscle biopsy.

1 These patients had intermittent stays at other departments: The total days of stay in hospital are stated (numbers in parentheses are days of stay in ICU solely). M: Male, F: Female, S: Survival, D: Death. NA: Noradrenaline, A: Adrenaline, IV: Intravenous, PO: Peroral, EN: Enteral, PN: Parenteral.

As specified in table 2, the included patients were, on average, moderately hyperglycemic at the time of the biopsy (Table 2) as well as 111±22 minutes prior to the biopsy (mean±SEM 9.1±0.8 mmol/l), and all received insulin therapy to control hyperglycemia during their ICU stay. All except patient 2 received insulin (1–10 IU per hour) at the time of the biopsy (Table 2), and the individual doses were the same 61±5 minutes prior to the biopsy. Patients 5 and 8 had diagnosed type 2 diabetes (T2D), whereas patient 11 likely had unrecognized T2D. Patient 10 had type 1 diabetes.

**Table 2:** ICU patient glucose concentrations and insulin doses.

Patient #	Blood glucose conc. at biopsy mmol/l	Insulin dose at biopsy IU/h	Notes
1	10.4	5.0	
2	7.0	0.0	
3	6.5	7.5	
4	8.5	8.0	
5	9.1	2.0	T2D
6	7.9	1.5	
7	8.3	2.5	
8	8.0	10.0	T2D
9	9.7	3.0	
10	14.2	5.5	T1D
11	9.3	10.0	T2D^1^
12	6.8	1.0	
**Mean±SEM**	**8.8±0.6**	**4.7±1.0**	

IU: International units, T1D: Type 1 diabetes, T2D: Type 2 diabetes.

1 Patient 11 likely had unrecognized T2D.

### Markers of systemic inflammation, proteins, metabolites and muscle cytokines

**Figure 1:**
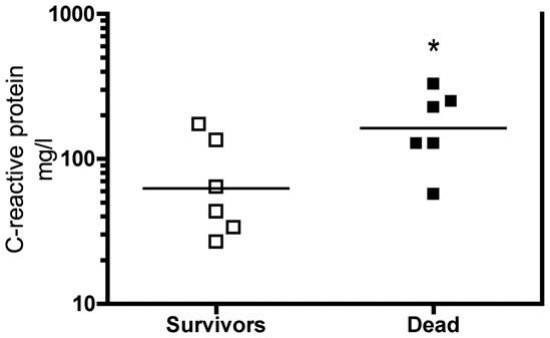
Higher plasma C-reactive protein levels in non-surviving than in surviving ICU patients. Data are fold change in individual values. Each square represents one patient (white: survivors, black: dead) and the bar represents the mean value. * p<0.05.

As shown in table 3, ICU patients had circulating levels of leukocytes, urea and creatinine approximately 2-fold above the normal range, whereas plasma CRP was increased by more than 10-fold compared to the upper normal range. Patients, who died had higher CRP levels than surviving patients (p<0.05, Figure 1). Serum albumin levels were below normal. Moreover, the levels of the pro-inflammatory cytokines MCP-1, TNF-α, IL-8 and IL-6 were approximately 4, 6, 52 and 170-fold higher in patients compared with control subjects (p = 0.05 for IL-8, p<0.05 for others, Table 3). This was also the case for the anti-inflammatory cytokine IL-10, which was more than 40-fold higher in patients (p<0.05).

**Table 3:** Circulating IGF-1, markers of systemic inflammation, proteins and metabolites.

Variable	Control subjects (n = 12)	ICU patients (n = 12)	% Difference
IGF-1, µg/l	132±14	76±14†	−43
***Inflammatory markers***			
Leukocytes, 10^9^/l	ND, normal range: 3.0–9.0	16.3±3.3	
CRP, mg/l	ND, normal <10	134±28	
TNF-α, pg/ml	3.9±0.5	27.3±8.4*	+605
IL-6, pg/ml	6.3±1.5	1096±366*	+17371
IL-8, pg/ml	3.8±0.5	201±91^1^	+5246
IL-10, pg/ml	13.9±2.7	607±262*	+4258
MCP-1, pg/ml	266±28	1438±213‡	+440
***Proteins and metabolites***			
Serum albumin, g/l	ND, normal range: 34–48	21.1±1.6	
Urea, mmol/l	ND, normal range: 3.1–8.1	16.9±4.3	
Creatinine, µmol/l	ND, normal range: 45–105	160±33	

All values were measured in plasma, except for leukocytes, which were determined in whole blood.

Values are means ± SEM. ND: Not determined.

*p<0.05,

†p<0.01,

‡p<0.001,

1 p = 0.05.

**Figure 2:**
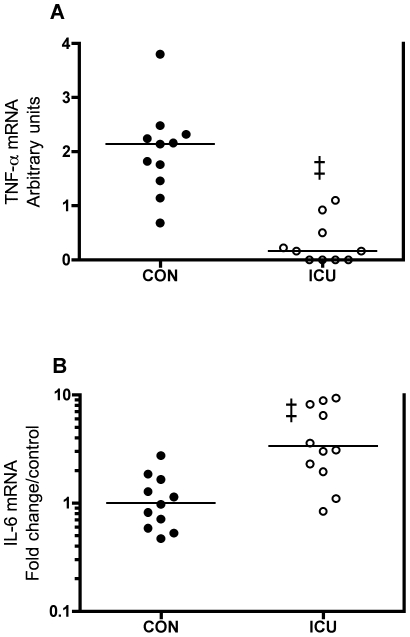
Lower TNF-α and higher IL-6 mRNA expression in muscle of critically ill patients. TNF-α (A) and IL-6 (B) mRNA expression in thigh skeletal muscle of critically ill intensive care unit (ICU) patients compared with controls (CON). As TNF-α mRNA was below the detection limit in some ICU patients (yielding 0 values), TNF-α mRNA was determined in quadruplicate and the median values for each group are reported in arbitrary units. IL-6 data are fold change in individual values relative to control mean. Each circle represents one subject (black: controls, white: ICU patients) and the bar represents the median TNF-α or the mean IL-6 value. ‡ p<0.001, Mann-Whitney U test for TNF-α and Welch's t-test for IL-6.

Contrary to the high levels of circulating pro-inflammatory cytokines in patients, the local muscle mRNA levels of TNF-α (−92%, p<0.001) and IL-1β (−56%, p<0.05) were lower in patients than in controls (Figure 2A and Figure S1 left). IL-1β protein was below our detection limit in plasma, whereas IL-8 and IL-10 mRNA were below our detection limit in muscle. Muscle MCP-1 mRNA was not different in patients vs. controls (Figure S1 right). Similar to plasma IL-6 protein, muscle IL-6 mRNA was 235% higher in patients compared with controls (p<0.001, Figure 2B).

### Plasma amino acids

**Figure 3:**
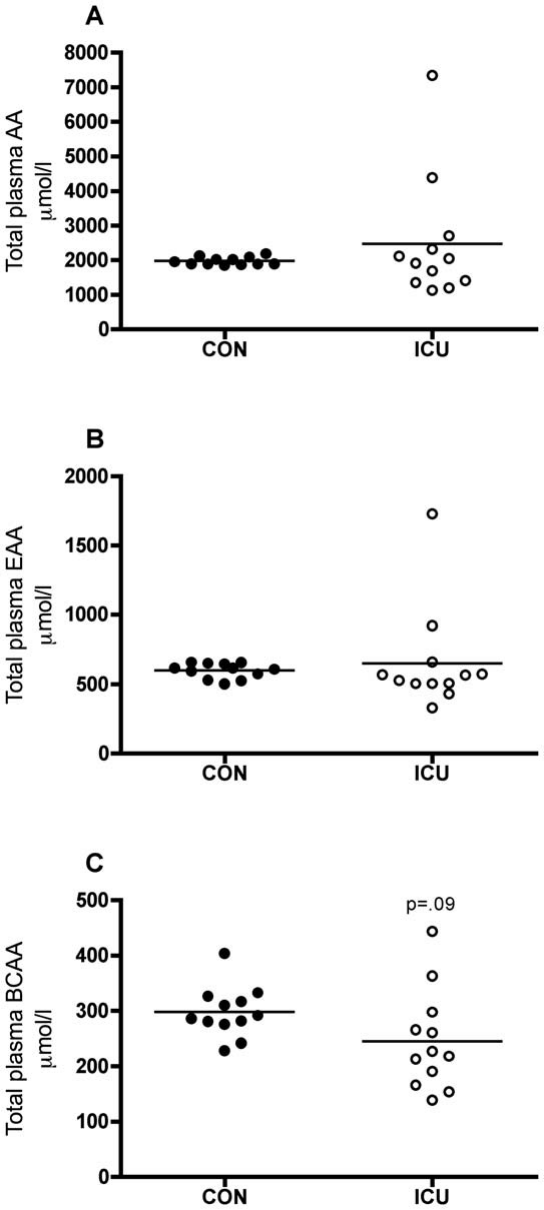
Lower branched chain amino acids in plasma of critically ill patients. Total plasma amino acids (AA) (A), essential amino acids (EAA) (B) and branched chain amino acids (BCAA) (C) in critically ill intensive care unit (ICU) patients compared with controls (CON). Each circle represents one subject (black: controls, white: ICU patients) and the bar represents the mean value.

Total plasma amino acids (AA) and essential AA (EAA) were not different between ICU patients and controls (Figure 3A and B), whereas branched chain AA (BCAA) tended to be 18% lower in patients (p = 0.09, Figure 3C). The BCAA leucine and valine were 22 (p = 0.08) and 21% (p<0.05) lower, respectively, in patients vs. controls, whereas phenylalanine and proline were 147 and 115% higher in patients (p<0.05, Table 4).

**Table 4:** Plasma amino acid concentrations (µmol/l).

Amino acid	Control subjects (n = 12)	ICU patients (n = 12)	P-value
Isoleucine	32±2	35±6	0.613
Leucine	89±5	69±9	***0.080***
Lysine	236±9	260±61	0.700
Methionine	29±2	57±14	***0.081***
Phenylalanine	36±1	90±16	**0.007**
Valine	177±7	141±12	**0.020**
Arginine	61±3	58±10	0.772
Histidine	61±2	66±11	0.652
Proline	165±8	355±67	**0.017**
Alanine	199±16	357±104	0.160
Glutamine	516±15	576±166	0.722
Glutamic acid	32±3	30±4	0.795
Glycine	203±16	254±55	0.392
Serine	84±5	58±9	**0.026**
Tyrosine	56±2	63±16	0.702
Total AA	1976±33	2469±511	0.356
EAA	599±16	652±106	0.635
BCAA	298±13	245±26	***0.086***

Values are means ± SEM. AA: amino acids, EAA: essential AA, BCAA: branched chain AA.

Total amino acids exclude asparagine, aspartic acid, cysteine, threonine and tryptophan.

Significant p-values are bold and tendency p-values (0.10>p>0.05) are bold italic.

### Akt-mTOR and Akt-GSK3β signaling

**Figure 4:**
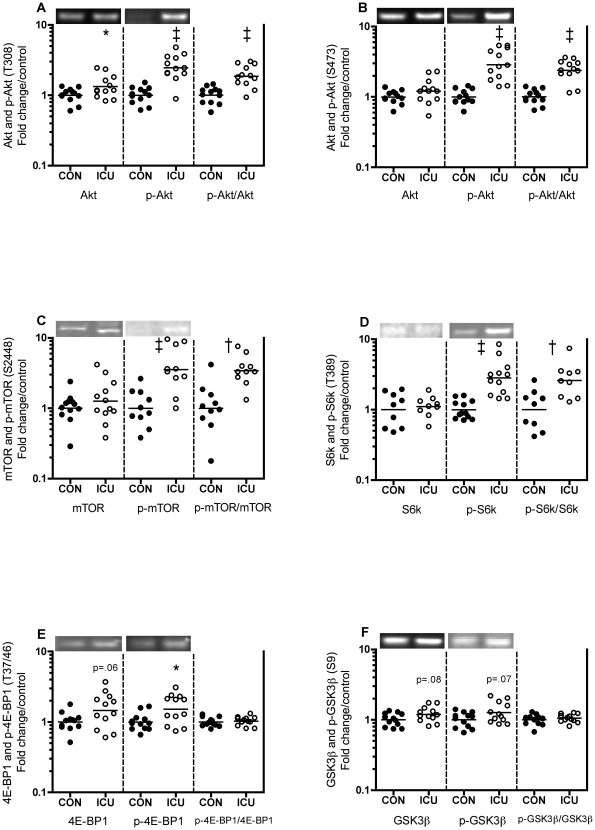
Substantially higher Akt-mTOR-S6k signaling in muscle of critically ill patients. Total (left), phosphorylated (middle) and phosphorylated/total ratios (right) of Akt (A and B), mTOR (C), S6k (D), 4E-BP1 (E) and GSK3β (F) proteins in thigh skeletal muscle of critically ill intensive care unit (ICU) patients compared with controls (CON). Representative blots are inserted. Phosphorylation status of Akt-mTOR-S6k were higher in ICU vs. CON. Data are fold change in individual values relative to control mean. Each circle represents one subject (black: controls, white: ICU patients) and the bar represents the mean value. * p<0.05, † p<0.01, ‡ p<0.001.

**Figure 5:**
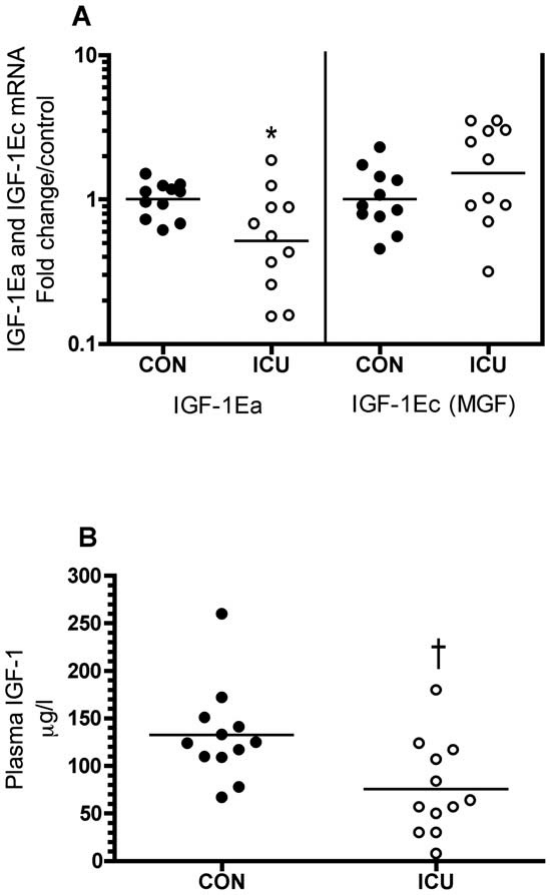
Lower muscle and circulating IGF-1 in critically ill patients. IGF-1Ea (A left) and IGF-1Ec (A right) mRNA expression in thigh skeletal muscle of critically ill intensive care unit (ICU) patients compared with controls (CON). mRNA data are fold change in individual values relative to control mean. Plasma IGF-1 levels in ICU patients vs. controls (B). Each circle represents one subject (black: controls, white: ICU patients) and the bar represents the mean value. * p<0.05, † p<0.01.

Phosphorylated Akt at threonine (T) 308 (p-Akt (T308)) and p-Akt (T308)/total Akt (Akt) were 146 and 86% higher in ICU patients compared with controls (p<0.001, Figure 4A middle and right). Akt was 33% higher in patients (p<0.05, Figure 4A left). Likewise, p-Akt (serine (S) 473) and p-Akt (S473)/Akt were 187 and 138% higher in patients (p<0.001, Figure 4B middle and right). P-mTOR (S2448) and p-mTOR (S2448)/mTOR were 254 and 243% higher in ICU patients vs. controls (p<0.001 and 0.01, Figure 4C middle and right), whereas p-mTOR (S2481) was below the detection limit. In ICU patients p-S6k (T389) and p-S6k/S6k were 183 and 160% higher compared with controls (p<0.001 and 0.01, Figure 4D middle and right). 4E-BP1 and p-4E-BP1 (T37/46) tended to be 46% and was 51% higher compared with controls (p = 0.06 and <0.05, Figure 4E left and middle), and, accordingly, p-4E-BP1/4E-BP1 was unchanged (Figure 4E right). GSK3β and p-GSK3β (S9) tended to be 19 and 26% higher compared with controls (p = 0.08 and 0.07, Figure 4F left and middle), and accordingly p-GSK3β/GSK3β was unchanged (Figure 4F right). Muscle IGF-1Ea mRNA and plasma IGF-1 protein were 48 and 43% lower in ICU patients vs. controls (p<0.05 and 0.01, Figure 5A left, Figure 5B and Table 3), while IGF-1Ec (aka mechano growth factor, MGF) was not different (+52%, p = 0.15, Figure 5A right).

### Correlation of insulin and Akt phosphorylation

**Figure 6:**
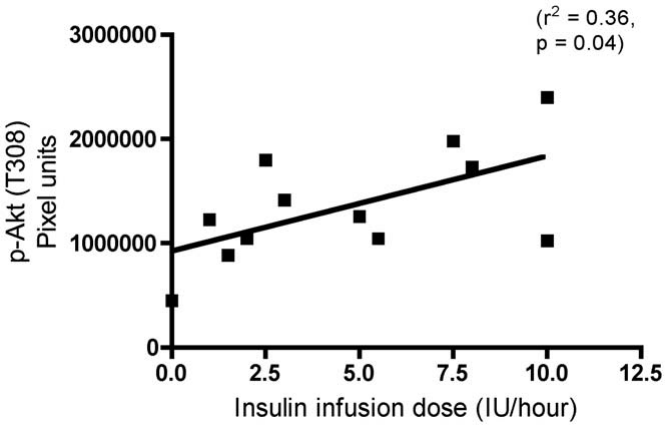
Correlation of insulin and Akt phosphorylation in critically ill patients. To estimate the role of insulin in the activation of Akt-mTOR-S6k signaling, we correlated insulin infusion doses at the time of the biopsy with Akt phosphorylation (p-Akt) at threonine (T) 308. A moderate correlation between insulin doses and p-Akt was demonstrated.

To estimate the role of exogenous insulin in the activation of the Akt-mTOR-S6k pathway, we correlated the insulin infusion dose at the time of the biopsy with phosphorylation of Akt at T308, which represents the upstream activation by 3-phosphoinositide dependent protein kinase 1 (PDK1). A moderate correlation (r^2^ = 0.36, p<0.05) between insulin doses and p-Akt (T308) was demonstrated (Figure 6).

### FoxO transcription factors, atrogin1 and MuRF1

**Figure 7:**
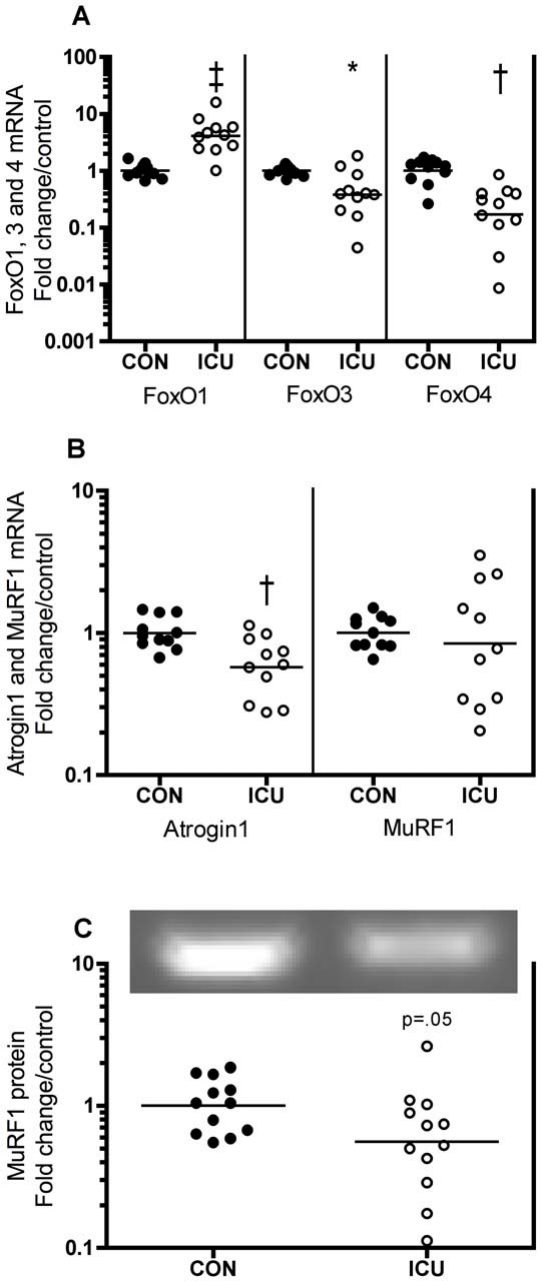
Divergent FoxO response and lower atrogene expression in muscle of critically ill patients. FoxO1, 3 and 4 (A left, middle and right), atrogin1 (B left) and MuRF1 (B right) mRNA expression and MuRF1 protein content (C) in thigh skeletal muscle of critically ill intensive care unit (ICU) patients compared with controls (CON). FoxO1 mRNA was higher, whereas FoxO3 and its downstream targets atrogin1 and MuRF1 were lower in ICU vs. CON. Representative MuRF1 protein blot is inserted. Data are fold change in individual values relative to control mean. Each circle represents one subject (black: controls, white: ICU patients) and the bar represents the mean value. * p<0.05, † p<0.01, ‡ p<0.001.

FoxO1 mRNA was 310% higher in ICU patients compared with controls (p<0.001, Figure 7A left), whereas FoxO3 and 4 mRNA were 62 and 83% lower compared with controls (p<0.05 and 0.01, Figure 7A middle and right). FoxO6 mRNA was below the detection limit. Unfortunately, we could not detect Foxo1, 3 and 4 proteins or any phosphorylation hereof. In ICU patients atrogin1 mRNA was 43% lower than controls (p<0.01, Figure 7B left). MuRF1 mRNA was similar to controls (−16%, p = 0.6, Figure 7B right), whereas MuRF1 protein was 44% lower compared with controls (p = 0.05, Figure 7C).

### Myostatin, GDF-11 and TGF-β1

**Figure 8:**
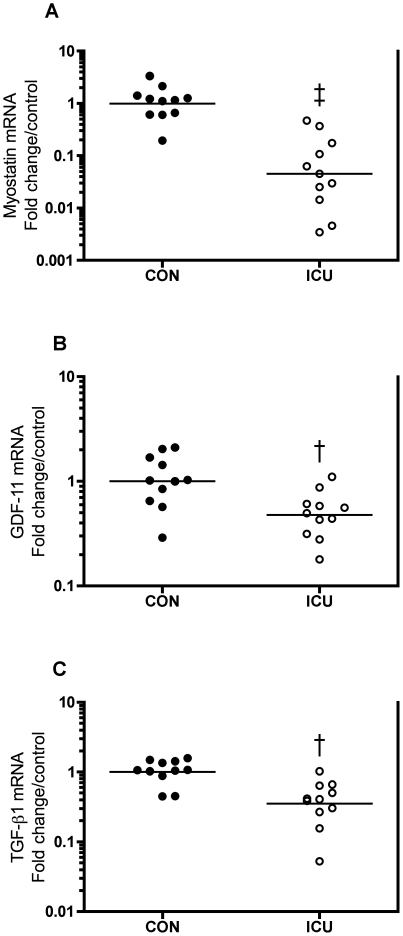
Lower myostatin, GDF-11 and TGF-β1 mRNA in muscle of in critically ill patients. Myostatin (A), GDF-11 (B) and TGF-β1 mRNA expression in thigh skeletal muscle of critically ill intensive care unit (ICU) patients compared with controls (CON). Data are fold change in individual values relative to control mean. Each circle represents one subject (black: controls, white: ICU patients) and the bar represents the mean value. † p<0.01, ‡ p<0.001.

mRNA levels of myostatin, (p<0.001), TGF-β1 and growth differentiation factor 11 (GDF-11) (p<0.01) were 95, 65 and 52% lower in ICU patients compared with controls (Figure 8A–C).

## Discussion

**Figure 9:**
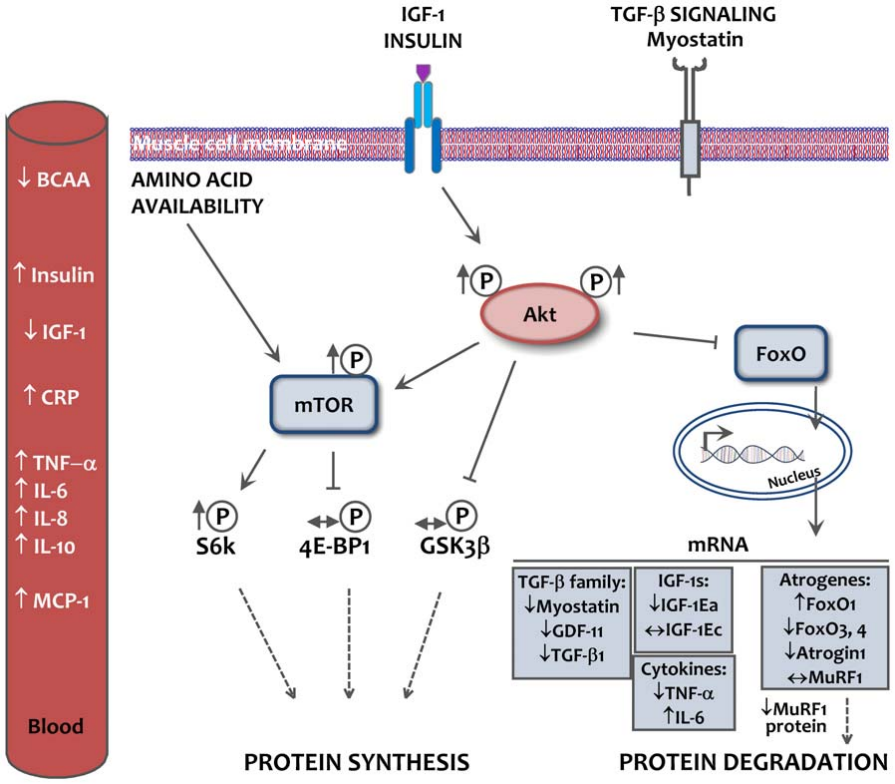
Muscle protein turnover signaling in critically ill ICU patients. Schematic overview of the results for circulating factors and muscle protein turnover signaling in ICU patients under the present study conditions. Arrows at the different targets show the levels in ICU patients compared with controls. Dotted lines depict that protein turnover was not determined. Abbreviations: 4E-BP1: eukaryotic translation initiation factor 4E binding protein 1, Akt: v-akt murine thymoma viral oncogene (aka PKB), BCAA: branched chain amino acids, CRP: C-reactive protein, GDF-11: growth differentiation factor 11, GSK3β: glycogen synthase kinase 3β, IGF-1: insulin-like growth factor 1, IL: interleukin, MCP-1: monocyte chemoattractant protein-1 (aka CCL2), mTOR: mammalian target of rapamycin, MuRF1: muscle ring finger 1, s6K: ribosomal protein S6 kinase, TGF-β: transforming growth factor β, TNF-α: tumor necrosis factor α.

We present for the first time muscle protein turnover signaling in critically ill patients, and we show signaling pathway activity in the direction of a stimulation of muscle protein synthesis and somewhat inhibited proteolysis. Specifically, we show unexpected higher Akt-mTOR-S6k signaling and lower FoxO3, atrogene and myostatin expression in ICU patients vs. controls (Figure 9).

### Akt-mTOR and Akt-GSK3β signaling

Surprisingly, in the muscles of systemically inflamed critically ill ICU patients (Tables 1 and 3) we show substantially higher phosphorylation of Akt and mTOR including its downstream targets S6k and 4E-BP1, whereas GSK3β tend to be lower than in healthy controls (Figure 4A–F). Upon phosphorylation, S6k is activated, enabling increased protein synthesis, whereas phosphorylation of 4E-BP1 releases it from the inhibitory complex with the translation initiation factor eIF4E, facilitating the binding of eIF4E to eIF4G and thereby promoting translation initiation [9], [10], [11]. An increase in both total and phosphorylated 4E-BP1 (Figure 4E) would thus probably cancel each other out. This higher Akt-mTOR-S6k signaling was unexpected, since phosphorylations at relevant sites of Akt, mTOR, 4E-BP, S6k and S6k's downstream substrate ribosomal protein S6 are decreased in muscles of different animal models of sepsis [10], [31], [32] and burn injury [33].

What may cause this higher Akt-mTOR-S6k signaling? IGF-1 and insulin are known activators of Akt-mTOR signaling [9], [10], [11], and Akt phosphorylation status was higher in postmortem rectus abdominis muscle biopsies of intensive insulin treated ICU patients compared with surgery patient controls [34], but no further downstream molecules were measured in that study. Since we in the present study show lower plasma IGF-1 and muscle IGF-1 mRNA in ICU patients (Figure 5A and B), neither systemic nor local IGF-1 could be the driving force in the activation of Akt-mTOR-S6k signaling. To further examine this question, we estimated the role of exogenous insulin by correlating insulin infusion dose at the time of the biopsy with Akt phosphorylated at T308, our most upstream target. We observed a moderate correlation (Figure 6) demonstrating responsiveness of Akt T308 phosphorylation in an insulin dose dependent fashion. Exogenous insulin dose may, however, be of limited value in the correlation with protein phosphorylation status, as ICU patients display signs of insulin resistance of varying degrees (Table 2) [35], and insulin sensitivity apparently increases with insulin therapy in critical illness [34], [36]. Accordingly, it is currently unclear if insulin therapy activates Akt-mTOR-S6k signaling in ICU patients under the present study conditions, and whether insulin induces muscle protein synthesis in critically ill ICU patients similar to what is seen in ICU burn patients [37].

Alternatively or additionally to insulin, an enhanced S6k phosphorylation could reflect an increased protein synthesis driven by the increased availability of intracellular amino acids resulting from increased proteolysis as shown in burn patients [38]. Amino acids, especially leucine, are known to stimulate skeletal muscle protein synthesis via both insulin-dependent and insulin-independent mechanisms [39]. Interestingly, S6k phosphorylation is increased in insulin-unstimulated rat burn injury [21], and we show a vastly higher S6k phosphorylation (Figure 4D). Since amino acids activate mTOR partly independent of insulin, and since mTOR phosphorylates S6k, the authors [21] suggested that increased amino acid availability could increase insulin-unstimulated protein synthesis through mTOR-S6k independent of Akt. However, it should be noted that the insulin-dependent mechanism of leucine-enhanced protein synthesis is associated with increased phosphorylation of S6k and 4E-BP1 [40] and that the stimulating effect of leucine on protein synthesis is impaired in animal sepsis [41]. Overall, in our fed ICU patients compared with post-absorptive controls we observe a very similar pattern of differences in plasma amino acid concentrations including lower leucine levels in patients (Table 4) as shown previously in post-absorptive septic patients compared with healthy controls [42]. Importantly, lower plasma amino acid concentrations do not necessarily equal lower muscle intracellular levels [43]. Specifically, lower plasma BCAA concentrations are not mirrored by low muscle levels [43] and in fact burn patients present with higher muscle leucine but similar plasma levels relative to healthy controls [38]. Accordingly, the possibility of mTOR specifically and general protein turnover signaling being affected by nutrients and amino acids should be considered.

Importantly, an increased Akt-mTOR signaling, as observed in atrophic COPD patients, may also represent an unsuccessful attempt trying to restore muscle mass [44], which agrees with ICU patient transcriptome data indicating a sustained protein synthesis similar to that in healthy controls in concert with a transcription program unable to compensate for enhanced protein breakdown [23], [45]. A dysfunctional muscle regenerative system is supported by a tendency (p = 0.09) in ICU patients to lower mRNA levels of tenascin-C (Figure S2), an apparent key regulator of muscle repair [46].

### FoxO transcription factors, atrogin1, MuRF1 and TNF-α

FoxO3 is required for and causes skeletal muscle atrophy through the ubiquitin-proteasome and autophagic-lysosomal proteolytic pathways [12], [13], [14], [47]. In several murine models of muscle loss FoxO1 mRNA is increased [17], [48], [49] including different sepsis models [22], [50], [51]. Here, we show a differing response of muscle FoxO1, 3 and 4 in critically ill patients. The amount of FoxO1 mRNA was more than 3-fold higher, whereas the amount of FoxO3 and 4 mRNAs were 60–80% lower in patients vs. controls (Figure 7A). Importantly, this divergent response of FoxO1, 3 and 4 suggests different functional roles for the specific FoxO members in human skeletal muscle. To our knowledge, we here present the first data on FoxO4 mRNA expression in human skeletal muscle, and we show an 83% lower expression in muscle of critically ill patients. This is in agreement with numerous animal studies, as FoxO4 mRNA was unchanged with corticosteroids and sepsis [49], [50], [51], but not all, since FoxO4 mRNA increased with glucocorticoids [48]. Activation of FoxO3 induces atrogin1 and MuRF1 transcription [12], [14], and these two ubiquitin ligases are also atrogenes [17]. To our surprise, atrogin1 mRNA and MuRF1 protein were lower in muscle of critically ill patients (Figure 7B–C), which is in contrast to their upregulation in several murine models of muscle atrophy [17] and the increased atrogin1 mRNA expression in septic ICU patients [23]. This discrepancy prompted us to determine two of the most responsive genes in that ICU patient study [23], IL-6 receptor (IL-6R) and actinin3, which were approximately 10 and 6-fold up- and downregulated, respectively. We found IL-6R to be unchanged, whereas actinin3 dramatically decreased by 97% (p<0.001, Figure S3), which demonstrate differences between the two studies. Of these differences, the fact that control subjects in the study by Fredriksson et al [23] were patients undergoing elective surgery is the most striking difference between the two studies.

Different roles of FoxO1 and FoxO3 in insulin and AMPK signaling have been suggested in previous in vitro non-muscle cell studies [52], [53] and in rat sepsis-induced muscle wasting [51]. Regarding different roles of FoxO isoforms in muscle atrophy, FoxO3 per se causes atrophy and is sufficient to induce atrogin1 and MuRF1 transcription [12], [14], whereas some studies [54], [55], but not all [56], show that FoxO1 is not sufficient to increase atrogin1 and MuRF1 mRNA expression in muscle cells. Insulin (or IGF-1) downregulates atrogin1 and to a lesser extent MuRF1 mRNA in vitro [57], and Akt activation mimics this by decreasing atrogin1 and MuRF1 mRNA expression and inhibits atrophy [12], [54], [58]. Accordingly, most of the available in vitro and animal-derived data on atrogin1 and MuRF1 expression are consistent with the observed downregulation of atrogin1 and MuRF1 being due to insulin administration, Akt phosphorylation and FoxO3 downregulation. Moreover, we have recently found a similar occurrence in human glucose-induced hyperinsulinemia showing decreased skeletal muscle atrogin1 and MuRF1 mRNA in concert with increased FoxO1 and unchanged FoxO3 mRNA (Nedergaard, Jespersen, Schjerling and Kjaer, unpublished) similar to results from rats [59]. Additionally, insulin downregulates TNF-α, but not IL-6 mRNA, in human skeletal muscle in vivo [60]. Accordingly, in the present study it seems likely that lower atrogene and TNF-α expression in ICU patients is regulated by the higher Akt phosphorylation and possibly insulin administration, but this remains to be proven in a more controlled set-up. Interestingly, similar to the present study, Greenhaff et al [61] in insulin-clamped healthy, young men, showed a relationship between increased serum insulin concentration and increased Akt and S6k phosphorylation as well as decreased atrogin1 protein levels, which, however, was disassociated with changes in muscle protein turnover. This may suggest that the magnitude of phosphorylation of these proteins under conditions of elevated insulin does not reflect muscle protein synthesis, at least in young, healthy individuals [61], even though insulin stimulates human muscle protein synthesis through increased nutritive flow and mTORC1-S6k signaling [62]. Of note, insulin (and IGF-1) resistance of protein breakdown is observed in septic and injured rats [63], [64], [65], and IGF-1 fails to prevent pro-inflammatory cytokine-induced myotube atrophy in vitro despite inactivation of FoxO3 and decreased atrogin1 [66].

### Myostatin, GDF-11 and TGF-β1

ICU patients showed a dramatic 95% lower myostatin and likewise lower GDF-11 and TGF-β1 mRNA levels compared with controls (Figure 8). To our knowledge, this is the first demonstration that GDF-11 mRNA is expressed in human skeletal muscle, and we show that GDF-11 responds similar to the 90% sequence-similar myostatin. Our finding of substantially higher Akt activation (Figure 4A and B) agrees well with the fact that Akt activation blocks TGF-β signaling and its atrophic effects in mice muscle [67]. Furthermore, the similar response of TGF-β1 and in particular GDF-11 is interesting, since negative regulators of muscle mass similar to myostatin exist [68], [69], and even though studies of a muscle-specific conditional GDF-11 knock-out mice indicate that GDF-11 does not regulate muscle size [70] TGF-β1 and other TGF-β superfamily members are candidate regulators of muscle mass.

### Limitations of the study

Overall, human studies in general and patient studies specifically entail limitations including human heterogeneity. That being said, most signaling pathways involved in skeletal muscle remodeling in general and in sepsis have so far been established by using rodents, and confirmation of observations from these models is required as to whether these pathways are physiologically valid and involved in humans [10], [71], an attempt that has been done seldom in ICU patients when it comes to skeletal muscle [23], [72]. Regardless of a probably increased systemic inflammation in non-surviving vs. surviving patients (Figure 1), a finding also seen in larger studies [73], [74], overall none of the differences in molecular signaling between controls and ICU patients were driven by any higher or lower expression or phosphorylation status in non-survivor vs. survivor patients. Despite the variations in the patients' diagnoses, ICU days at biopsy and clinical treatment (Table 1), our data show rather small variation indicating that the patient heterogeneity is of limited importance in the interpretation of the present results. This is supported by previous studies on heterogeneous ICU patients showing notable similarities in numerous biochemical and physiological parameters regardless of diagnosis and length of ICU stay [4], [75], [76], which indicate that the metabolic sequelae in critical illness could be a common response largely independent of admission diagnosis [4], [76]. This view is further substantiated by a recent microarray study on ICU patients receiving insulin and corticosteroid therapy showing a remarkably similar muscle transcript profile and the development of a specific and clear muscle phenotype in these heterogeneous patients [23], who are very similar to our patients with respect to diagnoses, age (mean±SEM 64±3 vs. 64±5 years) and ICU days at biopsy (7±3 vs. 6±2 days) (Table 1) [23].

Another limitation of our study is the lacking measures of preferably sequential changes in muscle fiber cross-sectional area (CSA) and muscle fractional protein synthesis and breakdown, as done previously in patients with burns [37], [38]. However, multiple biopsies from each patient were impossible due to their critical condition. The potential comparison between single time point muscle fiber CSAs of ICU patients and controls is, however, not without interpretational problems either. As detailed in table 1, 8 of 12 patients were current or previous abusers of alcohol, which in itself causes muscle atrophy [77], and some patients had prior to their ICU admission been hospitalized at other departments. Accordingly, the comparison of fiber CSAs between patients and controls would possibly show a lower mean fiber size of the patient group and indicate muscle atrophy. Importantly, this comparison will not, however, tell us whether this potential decrease in fiber area occurred before or during ICU admission, and, additionally, the considerable interpersonal variation in fiber size and number [78] will certainly limit the use of this comparison. Still, the ICU patients show some indications of muscle loss. The mean stay at the time of the biopsy was 6±2 days, i.e. within 10 days after ICU admission, where muscle atrophy is observed in some ICU patients [5], [6].

The patients had high CRP and IL-6 levels and low serum albumin levels (Table 3), which are part of the suggested diagnostic criteria for cachexia [3], acknowledging that CRP and IL-6 mainly are markers of inflammation. Despite these indices on muscle wasting it has to be acknowledged that we do not know if our patients experienced an active muscle loss or more stable state of atrophy at the time of the biopsy. A more stable state of atrophy could fit with our surprising observations of stimulated protein synthesis pathways and somewhat inhibited protein degradation pathways. Indirect evidence for muscle atrophy is the finding of a good agreement between the atrogene profile observed in several murine muscle atrophy models and the transcriptional response of ICU patients similar to our patients [17], [23]. On the other hand, the ICU patients under the present study conditions recapitulate a signaling pathway activity pattern, which in some animal models [79], [80], but not all [81], is related to the counteraction of disease-related muscle loss.

Finally, the posttranslational regulation of FoxO transcription factors by e.g. phosphorylation [82] should be recognized as a main regulatory event that was not examined in the present study. We could not detect any total or phosphorylated FoxO proteins, which may be due to FoxO protein content below our detection limit, since low endogenous FoxO protein expression is present in mouse and rat muscle cell lines [13], [15], [51] and rat skeletal muscle [14].

### Summary

In critically ill ICU patients compared with healthy controls, we show substantially higher Akt-mTOR-S6k signaling and lower FoxO3, atrogene and myostatin expression directed towards stimulated muscle protein synthesis and somewhat inhibited proteolysis. This is a surprising finding given the atrophic state that the patients most likely are in. The observed divergent response of the FoxO transcription factors suggests different functional roles for the specific FoxO members in human skeletal muscle. The lower FoxO3, atrogene and TGF-β family member expression can be explained by the higher Akt activation. The unexpected higher Akt-mTOR-S6k signaling may reflect a stimulating effect of insulin therapy on protein synthesis signaling, increased protein synthesis due to increased availability of intracellular amino acids resulting from increased proteolysis and/or an attempt to restore skeletal muscle mass. Importantly, it is currently unsolved whether the observed molecular responses reflect changes in muscle protein turnover and how they affect an apparent dysfunctional muscle remodeling machinery. Further, the present study suggests members of Akt-regulated pathways as proteins of interest in future studies of the molecular mechanisms of insulin on skeletal muscle protein metabolism in human critical illness. Due to the study design limitations in critical illness investigations, ex vivo muscle cell cultures from patient donors may be one approach to mechanistically clarify the role of hormones and nutrients on muscle mass regulating signaling. Despite the recent questioning of the intensity of insulin treatment of critical illness hyperglycemia [83], in a clinical perspective the effects of insulin on muscle protein metabolism should be considered and further investigated.

## Supporting Information

Figure S1 **Lower IL-1β mRNA expression in muscle of critically ill patients.** Interleukin-1β (IL-1β) (left) and monocyte chemoattractant protein-1 (MCP-1) (right) mRNA expression in thigh skeletal muscle of critically ill intensive care unit (ICU) patients compared with controls (CON). Data are fold change in individual values relative to control mean. Each circle represents one subject (black: controls, white: ICU patients) and the bar represents the mean value. * p<0.05.
(TIF)

Figure S2 **Lower tenascin-C mRNA expression in muscle of critically ill patients.** Tenascin-C mRNA expression in thigh skeletal muscle of critically ill intensive care unit (ICU) patients compared with controls (CON). Data are fold change in individual values relative to control mean. Each circle represents one subject (black: controls, white: ICU patients) and the bar represents the mean value.
(TIF)

Figure S3 **Lower actinin3 mRNA expression in muscle of critically ill patients.** IL-6 receptor (IL-6R) (left) and actinin3 (right) mRNA expression in thigh skeletal muscle of critically ill intensive care unit (ICU) patients compared with controls (CON). Data are fold change in individual values relative to control mean. Each circle represents one subject (black: controls, white: ICU patients) and the bar represents the mean value. ‡ p<0.001.
(TIF)

Table S1 **Primers used for RT-qPCR.** Abbreviations: RPLP0: acidic ribosomal protein P0, FoxO: Forkhead box O, MuRF1: muscle ring finger 1, IGF-1: insulin-like growth factor, GDF-11: growth differentiation factor 11, TGF-1β: transforming growth factor 1 beta, TNF-α: tumor necrosis factor alpha, IL: interleukin, IL-6R: IL-6 receptor, MCP-1: monocyte chemoattractant protein 1.
(DOC)

Table S2 **Antibodies used for Western blot.** Antibodies were diluted in 10% Odyssey blocking buffer (Li-Cor Biosciences) in distilled water with 0.1% v/v Tween 20. All total and phosphorylated protein pairs, except for mTOR, were detected simultaneously on the same membrane. NA: Not applicable.
(DOC)
