# Supplementary material for: Activated Protein Synthesis and Suppressed Protein Breakdown Signaling in Skeletal Muscle of Critically Ill Patients
Source: PLoS One. 2011 Mar 31;6(3):e18090. doi: 10.1371/journal.pone.0018090 (PMC3069050; doi:10.1371/journal.pone.0018090)
Supplement: Table S1 — Primers used for RT-qPCR. Abbreviations: RPLP0: acidic ribosomal protein P0, FoxO: Forkhead box O, MuRF1: muscle ring finger 1, IGF-1: insulin-like growth factor, GDF-11: growth differentiation factor 11, TGF-1β: transforming growth factor 1 beta, TNF-α: tumor necrosis factor alpha, IL: interleukin, IL-6R: IL-6 receptor, MCP-1: monocyte chemoattractant protein 1. (DOC) [file pone.0018090.s004.doc]

| **Table S1. Primers used for RT-qPCR** | | |
| --- | --- | --- |
| **Target** | **Sense primer** | **Antisense primer** |
| RPLP0 | GGAAACTCTGCATTCTCGCTTCCT | CCAGGACTCGTTTGTACCCGTTG |
| FoxO1 | GCCCAACCAAAGCTTCCCACAC | TGGACTGCTTCTCTCAGTTCCTGCT |
| FoxO3 | GCTGGGTGCCAGGCTGAAGG | TTGGCAAAGGGTTTTCTCTGTAGGT |
| FoxO4 | GATGAGGGCGAGGGACTGGA | TCCACATCTGAAGCAGGGGACA |
| FoxO6 | TACGCCGACCTCATCACCAAAG | GACAGGTTGTGCCGGATGGAGT |
| Atrogin1 | TGTTACCCAAGGAAAGAGCAGTATGGA | ACGGAGCAGCTCTCTGGGTTATTG |
| MuRF1 | TGGGGGAGCCACCTTCCTCT | ATGTTCTCAAAGCCCTGCTCTGTCT |
| IGF-1Ea | GACATGCCCAAGACCCAGAAGGA | CGGTGGCATGTCACTCTTCACTC |
| IGF-1Ec | GCCCCCATCTACCAACAAGAACAC | CGGTGGCATGTCACTCTTCACTC |
| Myostatin | TGCTGTAACCTTCCCAGGACCA | GCTCATCACAGTCAAGACCAAAATCC |
| GDF-11 | CCACCACCGAGACCGTCATTAG | GGCTGAAGTGAAAATGGCAGCA |
| TGF-β1 | GAGGTCACCCGCGTGCTAATG | CACGGGTTCAGGTACCGCTTCT |
| TNF-α | TTCCCCAGGGACCTCTCTCTAATC | GAGGGTTTGCTACAACATGGGCTAC |
| IL-1β | TCCAGGGACAGGATATGGAGCA | AGGCCCAAGGCCACAGGTATTT |
| IL-6 | GAGGCACTGGCAGAAAACAACC | CCTCAAACTCCAAAAGACCAGTGATG |
| IL-6R | CAGCTTATCTCAGGGGTGTG | AAGATGAAACGATGCAGTGG |
| IL-8 | CCACACTGCGCCAACACAGAAA | TTCTCCACAACCCTCTGCACCC |
| IL-10 | CGCTGTCATCGATTTCTTCCCTGT | TGGCTTTGTAGATGCCTTTCTCTTGG |
| MCP-1 | GCCCTTCTGTGCCTGCTGCT | GCAGGTGACTGGGGCATTGATT |
| TenascinC | CAACCATCACTGCCAAGTTCACAA | GGGGGTCGCCAGGTAAGGAG |
| Actinin3 | CCGAGACTGACACGACTGAGCAA | CAGCTCCTCGGGGGTGATGTAG |

Abbreviations: RPLP0: acidic ribosomal protein P0, FoxO: Forkhead box O, MuRF1: muscle ring finger 1, IGF-1: insulin-like growth factor, GDF-11: growth differentiation factor 11, TGF-1β: transforming growth factor 1 beta, TNF-α: tumor necrosis factor alpha, IL: interleukin, IL-6R: IL-6 receptor, MCP-1: monocyte chemoattractant protein 1.
