# Supplementary material for: Activated Protein Synthesis and Suppressed Protein Breakdown Signaling in Skeletal Muscle of Critically Ill Patients
Source: PLoS One. 2011 Mar 31;6(3):e18090. doi: 10.1371/journal.pone.0018090 (PMC3069050; doi:10.1371/journal.pone.0018090)
Supplement: Table S2 — Antibodies used for Western blot. Antibodies were diluted in 10% Odyssey blocking buffer (Li-Cor Biosciences) in distilled water with 0.1% v/v Tween 20. All total and phosphorylated protein pairs, except for mTOR, were detected simultaneously on the same membrane. NA: Not applicable. (DOC) [file pone.0018090.s005.doc]

| **Table S2. Antibodies used for Western blot** | | | | |
| --- | --- | --- | --- | --- |
| **Primary antibodies** | | | | |
| *Antigen* | *Manufacturer* | *Product #* | *Phosphorylation site* | *Dilution* |
| Akt | Cell Signaling Technology | 2920 | NA | 1:2,000 |
| p-Akt | Cell Signaling Technology | 2965 | T308 | 1:2,000 |
| p-Akt | Cell Signaling Technology | 4060 | S473 | 1:2,000 |
| mTOR | Cell Signaling Technology | 2983 | NA | 1:2,000 |
| p-mTOR | Cell Signaling Technology | 2971 | S2448 | 1:2,000 |
| p-mTOR | Cell Signaling Technology | 2974 | S2481 | 1:2,000 |
| S6k | Cell Signaling Technology | 9202 | NA | 1:2,000 |
| p-S6K | Cell Signaling Technology | 9206 | T389 | 1:2,000 |
| 4E-BP1 | Santa Cruz Biotechnology | sc-81149 | NA | 1:200 |
| p-4E-BP1 | Cell Signaling Technology | 2855 | T37/46 | 1:2,000 |
| GSK3β | Abcam | ab31826 | NA | 1:2,000 |
| p-GSK3β | Cell Signaling Technology | 9336 | S9 | 1:2,000 |
| MuRF1 | Abcam | ab4125 | NA | 1:5,000 |
| **Secondary antibodies** | | | | |
| *Antigen* | *Manufacturer* | *Product #* | *Conjugate* | *Dilution* |
| Anti-mouse | Invitrogen | A21057 | Alexa Fluor 680 | 1:10,000 |
| Anti-goat | Invitrogen | A21084 | Alexa Fluor 680 | 1:10,000 |
| Anti-rabbit | Pierce Biotechnology | 35571 | DyLight 800 | 1:10,000 |

Antibodies were diluted in 10% Odyssey blocking buffer (Li-Cor Biosciences) in distilled water with 0.1% v/v Tween 20. All total and phosphorylated protein pairs, except for mTOR, were detected simultaneously on the same membrane. NA: Not applicable.
